# Antitumor efficacy of multi-target *in situ* vaccinations with CpG oligodeoxynucleotides, anti-OX40, anti-PD1 antibodies, and aptamers

**DOI:** 10.7555/JBR.36.20220052

**Published:** 2022-11-28

**Authors:** Anastasia S. Proskurina, Vera S. Ruzanova, Genrikh S. Ritter, Yaroslav R. Efremov, Zakhar S. Mustafin, Sergey A. Lashin, Ekaterina A. Burakova, Alesya A. Fokina, Timofei S. Zatsepin, Dmitry A. Stetsenko, Olga Y. Leplina, Alexandr A. Ostanin, Elena R. Chernykh, Sergey S. Bogachev

**Affiliations:** 1 Institute of Cytology and Genetics, Siberian Branch of the Russian Academy of Sciences, Novosibirsk 630090, Russia; 2 Department of Natural Sciences, Novosibirsk State University, Novosibirsk 630090, Russia; 3 Department of Physics, Novosibirsk State University, Novosibirsk 630090, Russia; 4 Department of Chemistry, Moscow State University, Moscow 119991, Russia; 5 Research Institute of Fundamental and Clinical Immunology, Novosibirsk, 630099, Russia

**Keywords:** immunogenicity, Krebs-2 carcinoma, Lewis carcinoma, Ehrlich carcinoma, A20 B cell lymphoma, mesyl phosphoramidate

## Abstract

To overcome immune tolerance to cancer, the immune system needs to be exposed to a multi-target action intervention. Here, we investigated the activating effect of CpG oligodeoxynucleotides (ODNs), mesyl phosphoramidate CpG ODNs, anti-OX40 antibodies, and OX40 RNA aptamers on major populations of immunocompetent cells *ex vivo*. Comparative analysis of the antitumor effects of *in situ* vaccination with CpG ODNs and anti-OX40 antibodies, as well as several other combinations, such as mesyl phosphoramidate CpG ODNs and OX40 RNA aptamers, was conducted. Antibodies against programmed death 1 (PD1) checkpoint inhibitors or their corresponding PD1 DNA aptamers were also added to vaccination regimens for analytical purposes. Four scenarios were considered: a weakly immunogenic Krebs-2 carcinoma grafted in CBA mice; a moderately immunogenic Lewis carcinoma grafted in C57Black/6 mice; and an immunogenic A20 B cell lymphoma or an Ehrlich carcinoma grafted in BALB/c mice. Adding anti-PD1 antibodies (CpG+αOX40+αPD1) to *in situ* vaccinations boosts the antitumor effect. When to be used instead of antibodies, aptamers also possess antitumor activity, although this effect was less pronounced. The strongest effect across all the tumors was observed in highly immunogenic A20 B cell lymphoma and Ehrlich carcinoma.

## Introduction

Cancer is the second leading cause of deaths worldwide, accounting for nearly 10 million lives in 2020^[1]^. The development of anticancer treatments is extremely complex, although this is obviously medical imperative. To address this problem, it is necessary to develop an in-depth understanding of fundamental principles of tumor growth and the mechanisms involved in antitumor immunity. Cells in the immune system can recognize and eliminate tumors. In these instances, the antitumor response is triggered by dendritic cells (DCs) that process and present tumor-associated antigens to naive T cells. This in turn drives the proliferation of T cells and their differentiation to effector T cells and memory T cells that are capable of killing tumor cells^[2–3]^. However, tumor cells are efficiently eliminated only at the initial stages of malignant transformation. When there are clinical manifestations, tumors have already become insensitive to cytotoxic lymphocytes, and can actively suppress the immune system, thereby inducing tolerance to tumor antigens^[4–5]^. Therefore, immunotherapies designed to overcome tolerance and to activate antitumor immune responses are widely considered one of the promising strategies for cancer treatment.

Inadequate immune response is caused by tumor-induced immunosuppression and the phenomenon of T cell exhaustion. Immune suppression is related to an increased level/activity of various suppressor cells (*i.e.*, regulatory T cells, myeloid-derived suppressor cells, tolerogenic DCs, M2 macrophages, and suppressor fibroblasts) that inhibit different stages in the immune responses. Whereas, T cell exhaustion is related to an increased co-inhibitory molecular expression, and these interacting molecules cause T cell apoptosis and/or anergy^[5–6]^. Therefore, efforts made over the past two decades have focused predominantly on the developing approaches to overcome T cell exhaustion and reactivate immune responses. Indeed, blocking antibodies that target inhibitory checkpoint molecules (*e.g.*, cytotoxic T-lymphocyte-associated protein 4 [CTLA-4] and programmed cell death protein 1 [PD1]) have shown to be highly efficient in experimental studies and relatively effective in clinical trials involving patients with metastatic cancer^[7]^. Among the approaches that focus on suppressor cell elimination, the only method implemented in clinical practice is the low-dose cyclophosphamide treatment that eliminates suppressor/regulatory T cells^[8]^ and inhibits the suppressor activity of myeloid-derived suppressor cells^[9]^.

The relatively new antitumor immunity activation paradigm revolves around the idea of immune reprogramming. The immune reprogramming and overcoming immune tolerance during tumor growth require multi-targeting aspects of the immune system using combinations that aim to stimulate DCs, trigger signaling *via* T cell co-stimulatory receptors (*e.g.*, OX40, also known as CD134) and inhibit signaling *via* T cell co-inhibitory molecules (*e.g.*, PD1). The aforementioned cell types, receptors, and molecules, as well as their agonists and antagonists, are the focuses of new biotechnological developments for antitumor immunity activation known as *in situ* vaccinations. Local intratumoral immunotherapies, for example, using tumors as a source to personalize vaccines, while immunoactive substances are delivered directly into tumor tissues. The direct delivery of immunostimulants enables us to deliver high concentrations *in situ*, while using relatively low doses, as well as to explore various combinations of immunomodulators without risks of eliciting any systemic toxic response. Furthermore, the aforementioned approach triggers an effective immune response without the need to detect immunodominant epitopes in each particular case, *i.e.*, for a specific tumor type in a specific patient. Moreover, due to the migration capability of immune cells, intensive local tumor-specific T cell activation within the tumor growth site can elicit systemic antitumor immune responses that aim to destroy tumor cells within metastases^[10]^. Therefore, intratumoral administration of TLR4 agonist (G100) has been shown to enhance the efficacy of systemic administration of anti-PD1 monoclonal antibodies in the A20 B cell lymphoma model^[11]^. Intratumoral administration of agonistic OX40 antibodies in combination with CpG oligodeoxynucleotides (ODNs) has also been found to completely eliminate tumor foci and metastases in various tumor-graft models^[12]^. In this case, CpG ODNs increased the ability of antigen-presenting cells to induce OX40 receptor expression on T cells, while the administered agonistic OX40 antibodies activated the differentiation of naive T cells into effector T cells. Therefore, the following immunoactive factors are the key participants of antitumor immunity stimulation during *in situ* vaccination: DCs, T cells, OX40 receptors, PD1 receptors, agonist and antagonist antibodies against both receptor types, TLR9 and their agonists, CpG.

DCs are the specialised antigen-presenting cells capable of presenting tumor antigens to naive T cells and inducing the generation of antitumor cytotoxic lymphocytes and memory T cells^[13]^. Pro-inflammatory cytokines produced by DCs enhance expression of co-stimulatory molecules (required for the differentiation of effector T cells and the formation of immunological memory) on T cells. In combination with chemokines, they also ensure T cell recruitment into the tumor growth zone^[14]^ and induce the differentiation of myeloid-derived suppressor cells to inflammatory macrophages^[15]^. Furthermore, DCs exhibit a direct cytotoxic effect on tumor cells^[16]^. For these functions to be initiated, DCs need to become activated and mature; however, these processes are often suppressed in tumor microenvironment, and DCs exhibit tolerogenic properties^[17]^. Therefore, the activation of DCs is an essential component enhancing the effectiveness of the generation of cytotoxic lymphocytes and the infiltration of T cells into the tumor, weakening the immunosuppressive activity of tumor microenvironment.

CpG ODNs are TLR9 agonists activating the TLR9 signaling pathway to induce the maturation of DCs that produce pro-inflammatory cytokines, and to enhance the ability of DCs to stimulate T cell proliferation^[18]^. CpG ODNs can also inhibit the suppressant activity of myeloid-derived suppressor cells^[9]^. Monotherapy or combination therapy with SD-101, a class C CpG ODNs, are currently being investigated through clinical trials (NCT02927964, NCT02266147, NCT01745354, NCT02254772, and NCT02521870). Immunostimulatory CpG ODNs are considered to be among the most promising adjuvants in cancer immunotherapy due to their antitumor effect^[19–21]^. TLR9 receptors, on the other hand, are preferentially expressed by plasmacytoid DCs^[22]^. It has been shown that DCs generate from monocytes in the presence of IL-4, which also express TLR9 and can, therefore, be sensitive to the immunomodulatory action of CpG ODNs^[23–24]^. There are reports that stimulations of these DCs with CpG ODNs are accompanied by typical signs of DC maturation, including the increased expression of CD83, CD86, and human leukocyte antigen-DR isotype markers as well as the pronounced allostimulatory activity with respect to naive CD4^+^ T cells and their differentiation into interferon-γ (IFN-γ) secreting T helper type 1 cells^[23,25]^.

OX40 belongs to the family of tumor necrosis factor receptors, and appears on CD4^+^ and CD8^+^ T cells in response to T cell receptor stimulation. The ligand for OX40 (OX40L) is expressed on the activated antigen-presenting cells, including DCs, macrophages, and B cells. Activation of the OX40-OX40L signaling pathway enhances expansion, survival, and formation of memory T cells, as well as potentiates the functions of effector T cells^[26]^, while, the activation of this signaling pathway in regulatory T cells inhibits Foxp3 expression and reduces their suppressor activity^[27–29]^. In addition, agonistic OX40-specific antibodies exhibit antitumor activity in experimental studies and are being clinically tested^[30–32]^. However, weakly immunogenic tumors poorly respond to therapy with OX40 agonists because of the limited amount of tumor-infiltrating lymphocytes. Common drawbacks of monoclonal antibodies, directed against both inhibitory and stimulatory immune checkpoint molecules, and administered intravenously including non-specific toxic effects as well as inflammatory and autoimmune complications, are the most severe in the case of anti-CTLA-4 antibodies^[33]^.

One of the reasons for intrinsic tumor immune resistance is the expression of PDL1/L2 ligands on the surface of malignant cells. Ligands bind to the PD1 receptor and inhibit the activity of cytotoxic T cells, suppressing the antitumor immune response^[34–38]^. Blocking inhibitory checkpoints restores the functional activity of pre-existing cytotoxic T cells. While, this therapeutic type can be ineffective in the absence of cytotoxic lymphocytes (such tumors are known as "cold/non-inflamed" or "non-immune"), which explains why a number of patients have resistance to checkpoint inhibitors^[10]^. Immunotherapy aiming to generate cytotoxic lymphocytes is one of the ways to restore their population. The differentiation of naive T cells to cytotoxic lymphocytes requires the activation of co-stimulatory molecules (the so-called stimulatory checkpoint molecules), OX40 being one of those.

Blocking the mechanisms of peripheral tolerance, anergy, and exhaustion of T cells restores their function, resulting in regression of evasion of immune surveillance in cancer cells and tumor destruction^[39–44]^. Therefore, antitumor immune responses have two phases: the generation of cytotoxic lymphocytes related to the activation of naive T cells and their differentiation to effector cells (phase 1), and tumor destruction by effector T cells (phase 2). Anti-PD1/PDL1 monoclonal antibodies restore the effector phase of the immune response and, therefore, function only if there was also phase 1. Hence, the augmentation of the first and second phases of immune responses that use stimulatory checkpoint activators and that block of inhibitory checkpoint molecules should have a synergistic effect and should widen the range of tumors potentially sensitive to suppressors of inhibitory checkpoint molecules.

Aptamers are single-stranded oligonucleotides that are capable of binding to molecules of different nature with high affinity and specificity^[45]^. Essentially, aptamers are synthetic and functional equivalents of antibodies that are produced by *in vitro* selection from combinatorial libraries of oligonucleotides^[46]^. A large number of DNA or RNA aptamers targeting various cell surface antigens have been obtained and characterized^[47–48]^. Interactions between aptamers and their targets can exhibit both an induced and an inhibitory effect on the respective signaling pathways. The properties of aptamers are generally comparable with those of monoclonal antibodies; however, aptamers have a number of advantages due to their nucleic acid origin^[49–50]^. Aptamers can be chemically synthesized and modified, and are usually stable due to their pronounced secondary structures, and have no intrinsic toxicity. *In vivo* experiments showed that multimerized aptamers often exhibit the highest biological activity^[50–51]^.

Having a brief summary of the more up-to-date knowledge, we can hypothesize that the immune system is multi-faceted and needs to be exposed to a multi-target action for it to be reprogrammed and to respond more robustly to tumor growth and to overcome immune tolerance. The current study logically combines the results of two independent research steps aiming to justify and experimentally test the efficacy of a novel cancer immune therapy strategy, which involves *in situ* introduction of a combination of factors responsible for the stimulation of DCs, the activation of the signaling pathways through co-stimulatory receptors (OX40), and signaling inhibition through co-inhibitory molecules (PD1) of T cells. During the first step, we studied the activating effect of the selected active molecules in an *ex vivo* system on the major populations of immunocompetent cells involved in the development of antitumor immune response. This part of the study was conducted using human cell cultures, making it possible to examine the development of immune responses. In the long term, this is needed to proceed from experimental research to clinical studies. For assessing the activation of immunocompetent cells involved in the development of antitumor immune response, we chose active molecules that could be used in *in vivo* experiments (immunomodulatory CpG ODNs and agonistic OX40 aptamers). The commercially available anti-OX40 monoclonal antibodies were also used. During the second step, the resulting findings and their extrapolation to the mouse model were used to develop and test the experimental design making it possible to assess the antitumor effect of vaccination with a combination of selected active molecules on several types of mouse tumor models.

In the current study, the antitumor effect of multi-target synergistic treatment of experimentally induced tumors *via* *in situ* vaccination by intratumoral administration of activators of DCs, stimulatory checkpoint agonists, and inhibitory checkpoint antagonists were assessed. The efficacy of antitumor therapy was evaluated using four experimentally induced tumor models: Krebs-2 carcinoma, Lewis carcinoma, Ehrlich carcinoma, and A20 B cell lymphoma. The possibility of eliciting a cross-protective immune response was assessed for the models of A20 B cell lymphoma and Ehrlich carcinoma growing in the same animal.

## Materials and methods

### Oligodeoxynucleotides and aptamers sequences

Phosphorothioate CpG and non-CpG ODNs were obtained by standard means. Mesyl phosphoramidate CpG (μCpG) ODNs were prepared as described in Miroshnichenko *et al*^[52]^. To ensure longer circulation in the bloodstream, the μCpG ODNs were modified with palmitoyl group at the 5′-end of the ODNs at a maximum distance from the 3′-terminal palindrome, using a commercially available palmitoyl phosphoramidite (***Table 1***)^[53]^. To prevent a potential enzymatic cleavage of the 5′-terminal phosphodiester group, it was converted into mesyl phosphoramidate (μ-group)^[52]^.

**Table 1:** Oligodexynucleotide structures

ODNs and aptamers	Sequence (5′-3′)
Phosphorothioate CpG ODNs	TsCsGsAsAsCsGsTsTsCsGsAsAsCsGsTsTsCsGsAsAsCsGsTsTsCsGsAsAsT
Mesyl phosphoramidate CpG ODNs	Pal-TµCµGµAµAµCµGµTµTµCµGµAµAµCµGµTµTµCµGµAµAµCµGµTµTµCµGµAµAµT
Phosphorothioate non-CpG ODNs	TsGsCsAsAsGsCsTsTsGsCsAsAsGsCsTsTsGsCsAsAsGsCsTsTsGsCsAsAsT
Human OX40 aptamer	dGsdGdGdAdGdGdAdCdGdAdTdGdCdGdGrArArArArArArGrArAfCrAfCfUfUfCfCrGrAfUfUrArGrGrGfCfCfCrAfCfCfCfUrArAfCrGrGfCfCrGfCrArGdCdAdGdAdCdGdAdCdTdCdGdCdCdCdGsdA
Human template	TCGGGCGAGTCGTCTGTTTTTCGGGCGAGTCGTCTG
Mouse OX40 aptamer	dGsdGdGdAdGdGdAdCdGdAdTdGdCdGdGfCrArGfUfCfUrGfCrAfUfCrGfUrArGrGrArAfUfCrGfCfCrAfCfCrGfUrAfUrAfCfUfUfUfCfCfCrAfUdCdAdGdAdCdGdAdCdTdCdGdCdTdGdAdGdGdAdTdCdCdGdAdGsdA
Mouse template	TsCTCGGATCCTCAGCGAGT(TEG)(TEG)TCTCGGATCCTCAGCGAGsT
PD1 aptamer	GµACGATAGCGGTGACGGCACAGACGGCTACTGTACATCACGCCTCTCCCCCGTATGCCGCTTCCGTCCGTCGCTµCµ-Pal
ODNs: oligodeoxynucleotides; s: phosphorothioate linkage; µ: mesyl phosphoramidate linkage; d: deoxyribonucleotides; r: ribonucleotides; f: 2′-fluoro-2′-deoxyribonucleotides; TEG: tetraethylene glycol phosphate; Pal: palmitoylated abasic deoxynucleotide residue.	

We proposed to use OX40 aptamers as analogs of anti-OX40 antibodies. The sequences of agonistic human^[54]^ and mouse^[55]^ based aptamers against OX40 receptors were taken from the literature. OX40 aptamers were further stabilized by the insertion of two additional internucleotidic phosphorothioate groups at both 3′- and 5′-ends (***Table 1***). The human aptamers were intended for experiments with human cell culture, and we planned to use analogous mouse aptamers in experiments on *in situ* vaccination in mice. To mimic the anti-OX40 antibody structure, aptamers were dimerized in a head-to-tail fashion so that two identical receptor-binding domains were formed as a complementary duplex with oligonucleotide template (1 equiv. of the template per 2 equiv. of OX40 aptamer).

The two components were mixed, incubated for 5 minutes in a water bath heated to 95 ℃, and slowly cooled down to room temperature. A PD1 aptamer^[56]^ was also modified by incorporation of palmitoylated residue and stabilized against nuclease digestion by incorporation of three mesyl phosphoramidate linkages: two at the 3′-end and one more at the 5′-end (***Table 1***).

### *Ex vivo* experiments

Peripheral blood mononuclear cells (MNCs) were obtained by density gradient centrifugation (Ficoll-Paque, Sigma-Aldrich, USA) of heparinized whole blood samples from healthy volunteers (*n*=55), and informed consent was obtained from all donors. DCs were generated by culturing plastic-adherent MNC fraction in RPMI-1640 medium (Sigma-Aldrich) supplemented with 0.3 mg/mL L-glutamine, 5 mmol/L HEPES buffer, 100 µg/mL gentamicin, and 5% fetal calf serum (Sigma-Aldrich) in the presence of 40 ng/mL GM-CSF (Sigma-Aldrich) and 1000 U/mL IFN-α (Roferon-A, Roche, Switzerland) for three days at 37 ℃ and in a 5% CO_2_ atmosphere. To induce final maturation, DCs were incubated for 48 h in the presence of 10 µg/mL of lipopolysaccharide (LPS) from *E. coli* 0114:B4 (Sigma-Aldrich), 1 µg/mL CpG or 1 µg/mL µCpG. DCs without any maturation-promoting factor (DC_0_) added were also studied.

When performing an allogenic mixed leukocyte culture (allo-MLC) assay, MNCs from donors (0.1×10^6^ cells per well) were cultured for five days in the presence of immature DCs or DCs whose maturation took place in the presence of various maturation-promoting factors at a MNCs:DCs ratio of 10:1. After culturing for 48 h, 1 µg/mL purified anti-human CD134 antibodies (αOX40) (Biolegend, USA) or 5 µg/mL OX40 RNA aptamers were added. MNCs cultured in the presence of DCs without any subsequent addition of antibodies or aptamers were used as control.

On day 5, the count of OX40^+^CD8^+^ or CD8^+^CD107a^+^ Т cells in allo-MLC was measured by flow cytometry using APC anti-human CD134 (OX-40), FITC anti-human CD8, and APC anti-human CD107a antibodies (Biolegend).

The ability of DCs to elicit a proliferative response of T cells in allo-MLC is an integral parameter of the functional activity of DCs, since it is associated with the maturation state of DCs, the expression of Human Leukocyte Antigens and co-stimulatory molecules, as well as the range and level of cytokines produced by them. The proliferative response was assessed radiometrically on day 5 according to the incorporation of ^3^H-thymidine (37 kBq/well) added 18 h before the culturing was completed.

The cytokine production level (pg/mL) was measured by flow fluorometry on an automated double-beam laser analyzer using commercial Human Cytokine 8-plex (IL-2, IL-4, IL-6, IL-8, IL-10, GM-CSF, IFN-γ, and TNF-α) test kits (Bio-Plex Protein Assay System, Bio-Rad, USA) in accordance with the manufacturer's instructions.

### Dendritic cell activation

DC activation by synthetic CpG ODNs has been described thoroughly in our earlier study^[57]^. The activating DCs was also investigated for different compounds, *i.e.*, LPS, CpG ODNs (CpG and µCpG), dsDNA, and azoximer bromide. CpG ODNs were the most effective and were therefore used for further studies. CpG ODNs enhanced allostimulatory activity in human myeloid DCs. This was related to the maturation of DCs induced by CpG ODNs, which was confirmed by a decrease of CD14^+^ cells and simultaneously increases of CD83^+^ and CD86^+^ cells. These effects were more pronounced for µCpG than for CpG.

### Experimental animals

Five- to eight-month-old female C57BL/6 mice, three- to five-month-old male CBA/Lac mice, and two- to three-month-old female BALB/c mice (weight, 18–24 g) were bred at the Common Use Center Vivarium for Conventional Animals of the Institute of Cytology and Genetics of the Siberian Branch of the Russian Academy of Sciences (Novosibirsk, Russian Federation). Animals were kept in groups of 6–10 mice per cage with free access to food and water.

All animal experiments were performed in accordance with the European Convention for the Protection of Vertebrate Animals used for Experimental and other Scientific Purposes, and the protocol was approved by the Interinstitutional Bioethics Commission of the Institute of Cytology and Genetics of the Siberian Branch of the Russian Academy of Sciences (Protocol N 48/4 from 18.03.2019). Tumor burden did not exceed the recommended dimensions (*i.e.*, a maximum allowable diameter was 14 mm); animals were sacrificed using the method of cervical dislocation.

### Tumor models

Krebs-2 carcinoma, Lewis lung carcinoma, A20 B cell lymphoma, and Ehrlich adenocarcinoma were used as mouse tumor models. Krebs-2 carcinoma, Lewis lung carcinoma, and Ehrlich adenocarcinoma cells were obtained from the cell depository of the Institute of Cytology and Genetics, SB RAS (Novosibirsk, Russia). The A20 cell line was provided by the Laboratory of Immunogenetics at the Institute of Molecular and Cellular Biology, SB RAS (Novosibirsk, Russia).

A corresponding line of mice was chosen for each tumor: Krebs-2 carcinoma, CBA/Lac; Lewis lung carcinoma, C57BL/6; A20 lymphoma and Ehrlich adenocarcinoma, BALB/c.

To produce solid tumors, mice were intramuscularly engrafted with 1×10^6^–5×10^6^ tumor cells diluted in 0.2 mL of physiological saline solution into the hind paws. Solid tumors were measured with calipers, and tumor volume was calculated as follows: volume = length × width × height.

To produce ascites tumors, Ehrlich adenocarcinoma cells were intraperitoneally inoculated to BALB/c mice (5×10^6^ cells in 0.2 mL saline per mouse). The weight of ascites tumor was determined during an autopsy of the animals.

To produce two heterologous tumors (*i.e.*, A20 lymphoma and Ehrlich adenocarcinoma) growing simultaneously in one organism, the animals were first engrafted with A20 lymphoma cells (5×10^6^) and then, 10 days later, with Ehrlich adenocarcinoma cells (1×10^6^).

### Experimental use of technology for *in vivo* vaccinations in mice

Mice were engrafted with tumor cells of various model tumors. The volume of tumors at therapy initiation is shown in the figure legends. According to the respective study groups, mice were treated as follows: 50 µg of CpG, µCpG or non-CpG ODNs; 4 µg anti-OX40 monoclonal antibodies (CD134, clone OX86, 559861, BD Pharmingen, USA); 50 µg anti-PD1 monoclonal antibodies (CD279, clone RMP1-14, 114114, BioLegend); 100 pmol OX40 aptamers and 32 µg PD1 aptamers were injected intratumorally into the right paw in the experiments with two solid tumors. The tumor in the right paw is referred to as "treated tumor"; the tumor in the left paw is as "untreated tumor".

In the experiments with solid and ascites tumors, injections were made intratumorally into the solid tumor. In the experiments with two solid tumors (A20 lymphoma and Ehrlich adenocarcinoma), injections were made into the front paw, intratumorally at the growth site of Ehrlich adenocarcinoma. One of the ODNs, anti-OX40 and anti-PD1 antibodies or OX40 and PD1 aptamers were administered three times every other day. Control animals were treated with saline according to the same regimen.

### Immunogenicity assessment of* in situ* vaccination regimens

Immunogenicity assessment was performed using the A20 lymphoma model. The immunogenicity index (IND) took into account the ratio of treated and untreated tumor volumes on the selected day of measurements and was described by Ruzanova *et al*^[58]^. It was calculated as follows: IND = (V_untr_ − V_tr_) / V_untr_, where V_untr_ represents untreated tumor volume, V_tr_ treated tumor volume. The index was averaged for all animals in group. If IND is negative, then untreated tumor is smaller than treated tumor. If the IND number is close to zero, then the untreated tumor and treated tumor are similar in size. If IND >0.9, the untreated tumor is at least larger by an order of magnitude than the treated tumor.

### Statistical analysis

Statistical data were processed using the Statistica (version 8) software. The validity of differences was evaluated using the Mann-Whitney *U* test or Wilcoxon matched-pairs test. Comparison of the number of animals with tumor was performed using the Fisher's exact test. The Kruskal-Wallis test was used to determine whether or not there was a statistically significant difference in tumor size between groups and then the multiple comparison correction of mean ranks for all groups (Dunn's test) were conducted. The survival of mice was analyzed using the Kaplan-Meier method*.* Statistical method and significance level are indicated in each case in the text or in the figure legends.

## Results

### Naive T cell differentiation into cytotoxic T cells

**Figure 1:**
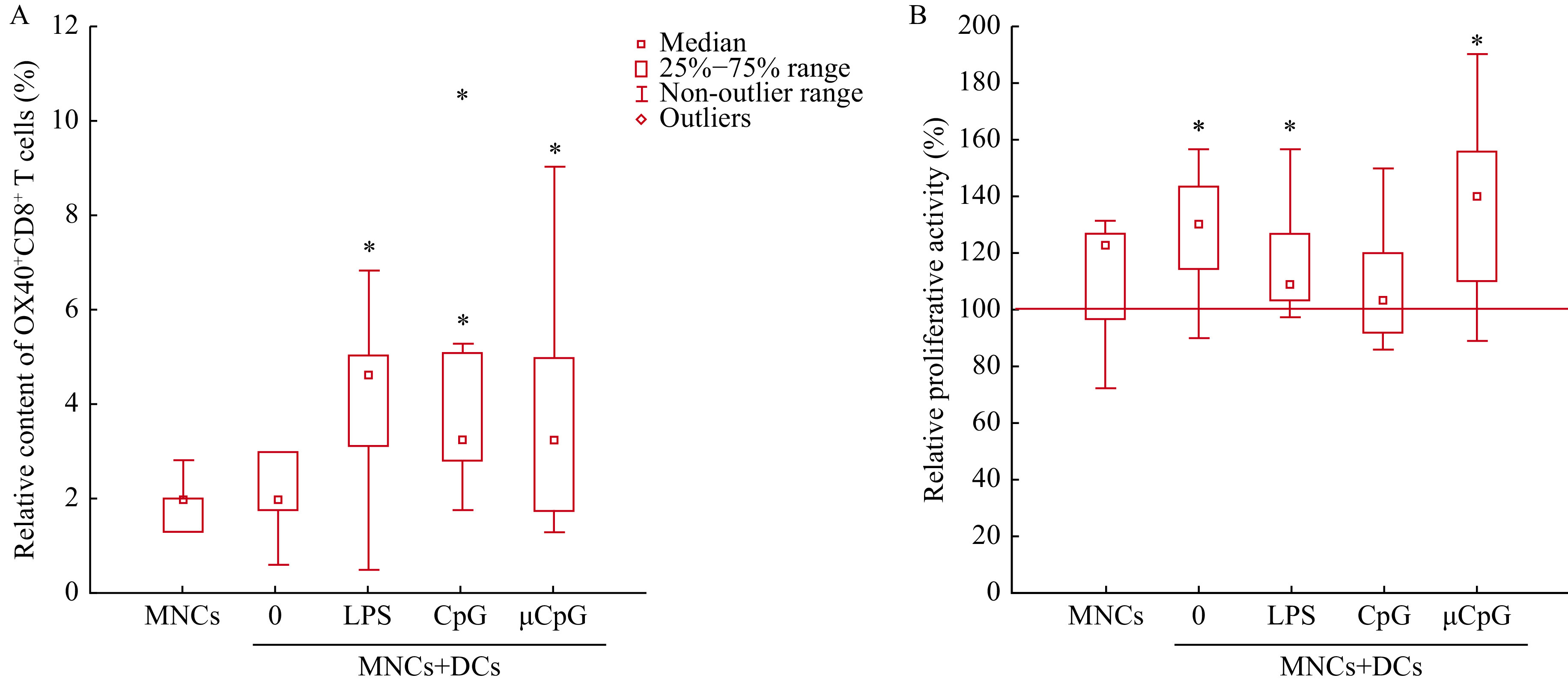
Effects of different activators of dendritic cells on their ability to stimulate the functional activity of cytotoxic T cells.

The effect of inducers on the ability of DCs to induce OX40 expression in the allo-MLC subpopulation of CD3^+^CD8^+^ T cells was assessed. LPS, a ligand of TLR4, was used as a positive control; however, because of its pyrogenicity, LPS cannot be used in clinical trials. The count of OX40^+^CD8^+^ T cells increased significantly in the presence of DCs that had been generated with CpG and µCpG (at a level similar to the case when LPS was used), compared with MNCs cultured without DCs (*P*<0.05, ***Fig. 1A***).

It is known that the interaction of agonistic monoclonal antibodies with OX40 receptors is a condition that is required to increase survival and to enhance the cytotoxic functions of effector T cells. One of the manifestations of this enhancement is that the proliferative activity of T cells in allo-MLC increases. In allo-MLC with DCs generated using the inducers (LPS, CpG or µCpG) or without it, the proliferative response of T cells in the presence of anti-OX40 agonistic monoclonal antibodies tends to increase, compared with MNCs cultured in the presence of same DCs without anti-OX40 antibodies assumed to be 100% (*P*<0.05, ***Fig. 1B***).

### Biological properties of anti-OX40 antibodies and ОХ40 RNA aptamers on proliferation, cytotoxic potential, and cytokine secretion of T cells

**Figure 2:**
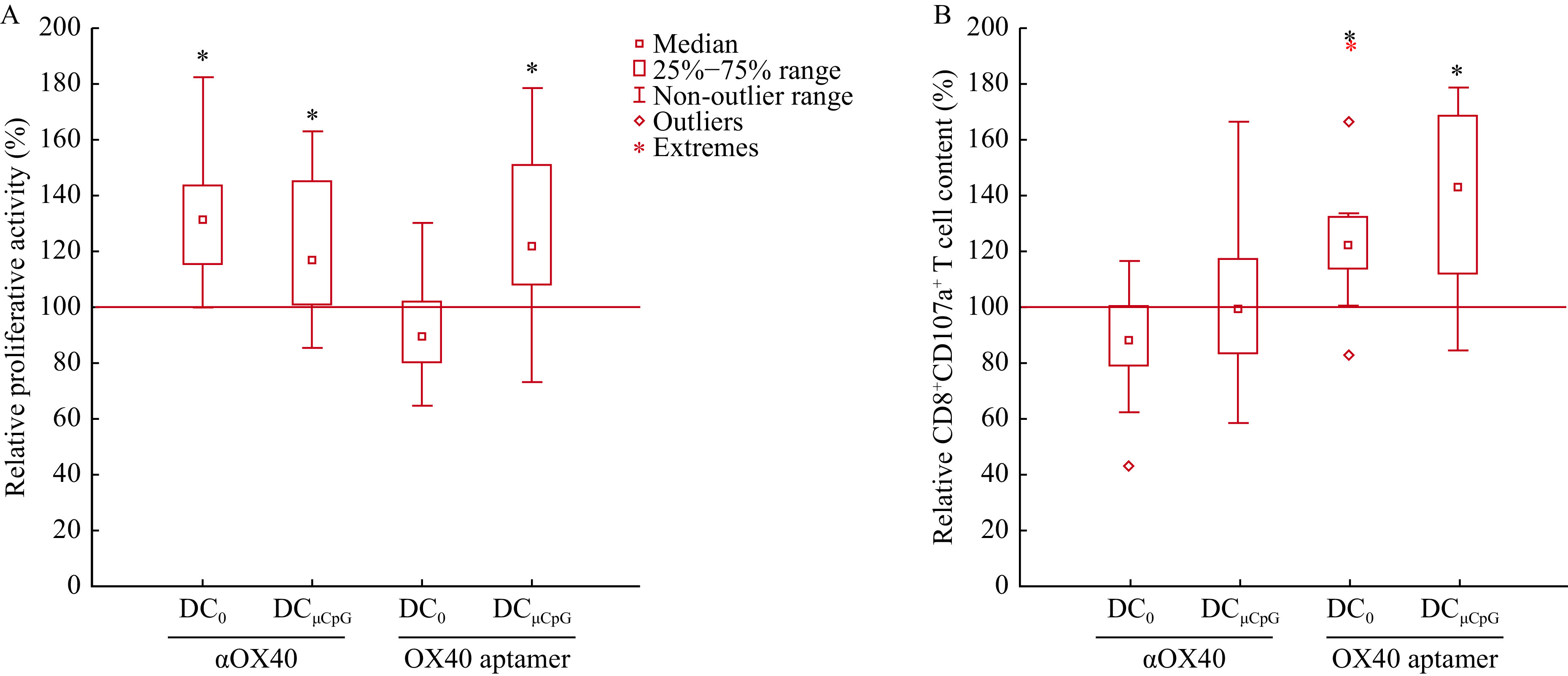
Biological activities of ОХ40 aptamers and αOX40.

ОХ40 aptamers, in the complexes with a dimeric matrix and with an extra-long insertion of two Spacer 18 residues, were synthesized and their biological activity was assessed and compared with agonistic monoclonal anti-OX40 antibodies, which were used as a positive control. In allo-MLC, the proliferative response of T cells exposured to DCs maturing in the presence of μCpG (DC_μCpG_) increased significantly in the presence of both αOX40 and ОХ40 aptamers, compared with that in the absence of antibodies or aptamers assumed to be 100% (*P*<0.05, ***Fig. 2A***). Additionally, ОХ40 aptamers stimulated the generation of cytotoxic CD8^+^CD107a^+^ T cells in allo-MLC in the presence of both immature and mature DCs, compared with that in the absence of aptamers assumed to be 100% (*P*<0.05, ***Fig. 2B***).

**Figure 3:**
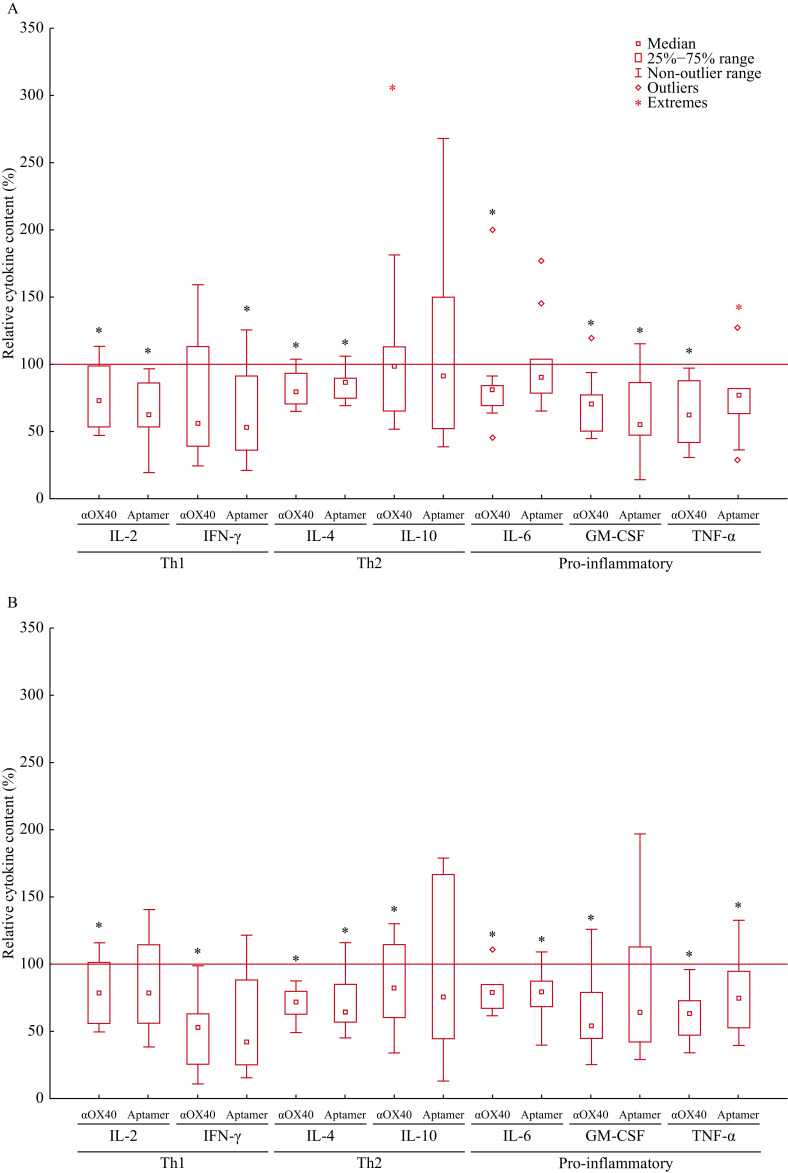
Cytokine production in allogenic mixed leukocyte culture.

Aptamer that effects on the production of cytokines by T helper type 1 cells (IL-2, IFN-γ), T helper type 2 cells (IL-4, IL-10), and pro-inflammatory cytokines (IL-6, GM-CSF, TNF-α) secreted in allo-MLC were also assessed. The results showed that the production of these cytokines was significantly reduced in the presence of both αOX40 and ОХ40 aptamers, compared with that in the absence of antibodies or aptamers assumed to be 100% (*P*<0.05). A decline in cytokine secretion function of T cells was detected in the presence of both immature DCs (***Fig. 3A***) and mature DCs stimulated by μCpG (***Fig. 3B***).

In general, the results indicated that the synthesized OX40 aptamers exhibited a selective activity, which consists of the stimulation of proliferation and the cytotoxic potential of T cells in allo-MLC in combination with weakening their cytokine secretion function. Biological properties of the synthesized OX40 aptamer complexes in the *ex vivo* system were comparable to those of the agonistic monoclonal anti-OX40 antibodies.

These findings infer that the multi-target action of active molecules, characterized previously in *ex vivo* experiments on the immune system of experimental animals with tumor graft models, provides insight into the actual therapeutic potential *in situ* vaccination modality.

### Comparative assessment of *in situ* vaccination antitumor effects with a modified oligodeoxynucleotides as a dendritic cell activator

*In situ* vaccination injections of different ODNs, anti-OX40, and anti-PD1 antibodies were performed. It was expected that CpG ODNs would increase the degree of DC activation, and that antibodies directed against checkpoint molecules would reduce innate immune suppression by stromal components in developing grafts^[12]^. Tumor models, including weakly immunogenic Krebs-2 carcinoma, moderately immunogenic Lewis carcinoma, and immunogenic A20 lymphoma, were grafted intramuscularly in two hind paws of mice. For the *in* *situ* vaccination protocol, only one paw was treated and the graft growth rate was evaluated.

**Figure 4:**
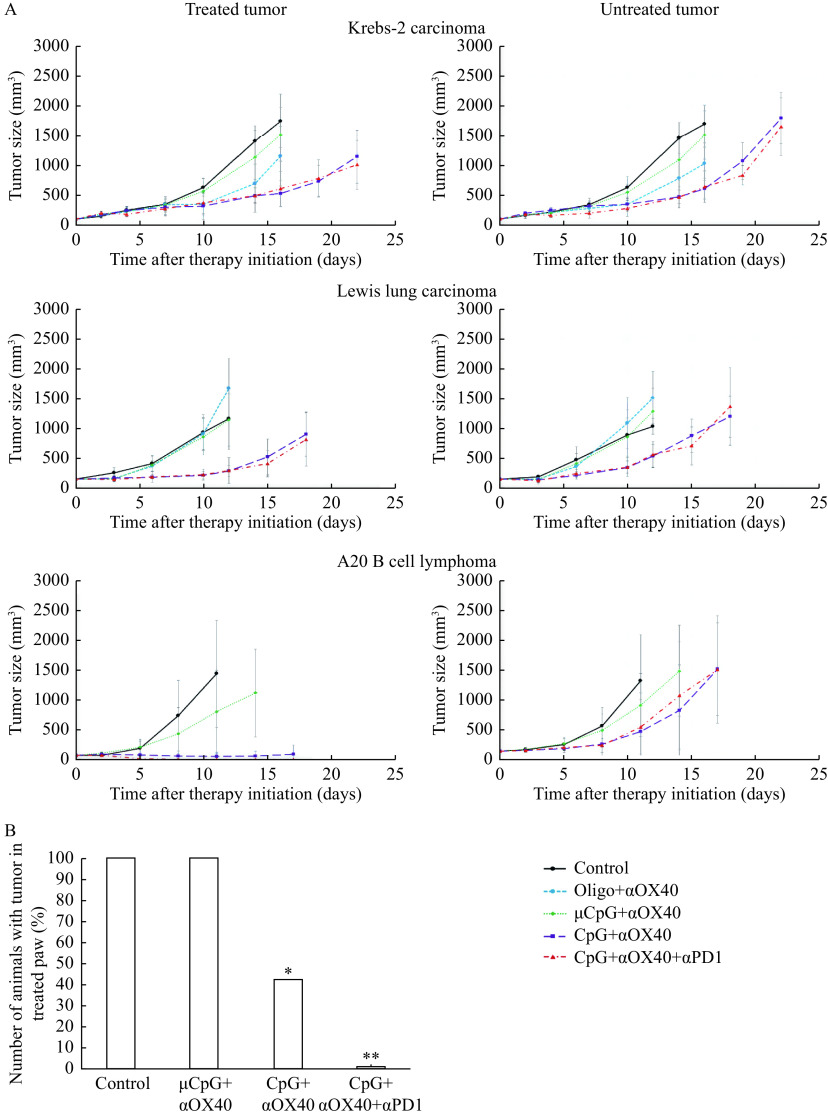
Efficacy of *in situ* vaccination with different ODNs, αOX40, and αPD1 for the Krebs-2, Lewis and A20 tumor models.

The use of anti-OX40 with either phosphorothioate non-CpG ODNs or µCpG for *in situ* vaccination had no significant impact on tumor growth, compared with the control (*P*˃0.05). The growth rate of tumors in the corresponding mouse groups was similar to the control group (***Fig. 4A***). The use of traditional phosphorothioate CpG was the most effective for all tumor therapy groups. For both Krebs-2 and Lewis carcinoma, we observed delays in transplanted graft growth, compared with control mice (*P*<0.01). Among all the tumors, the strongest effect of *in situ* vaccination with CpG and αOX40 was observed for the highly immunogenic A20 lymphoma. Almost complete tumor regression was observed at the treatment site (*P*<0.01). The growth rate of the treated graft was lower than that of the distant graft, with the distant graft growing slower than that in the control group, but these differences were not statistically significant (*P*˃0.05, ***Fig. 4A***).

Injections of anti-PD1 antibodies as part of the treatment had no additional significant effect on reducing the tumor growth rate (*P*˃0.05 between CpG+αOX40 and CpG+αOX40+αPD1 groups) (***Fig. 4A***). For A20 lymphoma, the administration of αPD1 showed a small tendency towards increasing the efficacy of *in situ* vaccination: on day 20 after treatment initiation the CpG+αOX40+αPD1 group contained no animals having a tumor at the treatment site, while their percentage in the CpG+αOX40 group was 43% (***Fig. 4B***).

The analysis and the findings reported by Ruzanova *et al*^[58]^ demonstrated that *in situ* vaccination was apparently applicable and highly efficacious only for immunogenic tumors. This finding enabled us to adjust the study design and to use only immunogenic experimentally induced tumors as tumor models in the subsequent studies, aiming to characterize the antitumor efficacy of aptamers. The results also demonstrated that *in vivo* antitumor activity of µCpG was much less than that of CpG, and was therefore excluded from further analysis.

### Antitumor efficacy of *in situ* vaccination with CpG oligodeoxynucleotides, anti-OX40 and anti-PD1 antibodies, and aptamers in the A20 lymphoma model

It has been shown previously that agonistic OX40 RNA aptamers and antagonistic PD1 DNA aptamers can stimulate (or block) afferent signaling pathways in T cells, which is accompanied by an activation of effector T cell proliferation and an increase in their survival^[54–56,59]^. Comparative analysis of the following regimens of *in situ* vaccination was performed: control mice, CpG+αOX40, CpG+OX40 aptamer, CpG+αOX40+αPD1, and CpG+OX40 aptamer+PD1 aptamer. The antitumor effect of injection of CpG ODNs alone was additionally assessed through experimentation.

**Figure 5:**
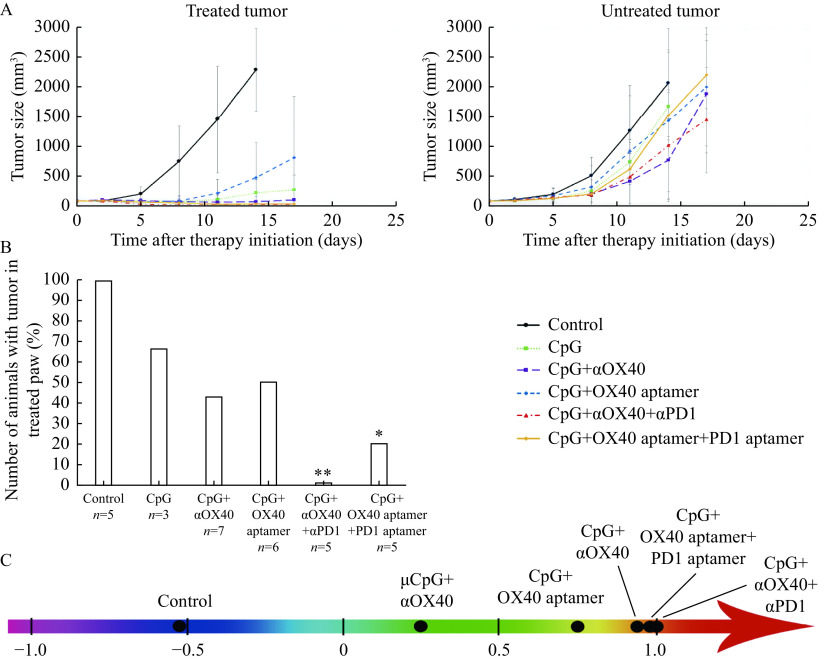
Efficacy of *in situ* vaccination with OX40 and PD1 aptamers for the A20 lymphoma model.

CpG injected alone had a nonsignificant antitumor effect (*P*˃0.05). The tumor at the treatment site completely regressed in one out of three mice in this group. The average tumor volume at the treatment site on day 17 was three times larger in this group, compared with mice receiving CpG+αOX40 injections (***Fig. 5A***). The efficacy of vaccination with CpG+OX40 aptamer was also comparable with the monotherapy with CpG, thereby suggesting that the aptamers alone had no effect (***Fig. 5A*** and ***B***).

The addition of anti-PD1 antibodies or aptamers significantly increased the antitumor efficacy of *in situ* vaccination, compared with the control (*P*<0.05). In the CpG+OX40 aptamer+PD1 aptamer group, the percentage of animals having a tumor at the treatment site was 2.5 times smaller than that in the CpG+OX40 aptamer group (***Fig. 5B***), while the average tumor size on day 20 was 30 times smaller, compared with the group that received no PD1 aptamers (***Fig. 5A***). The CpG+αOX40+αPD1 group contained no mice having a tumor at the treatment site, while the percentage of these animals in the CpG+αOX40 group was 43% (***Fig. 5B***).

Among the studied groups, the maximum antitumor activity was revealed for the CpG+αOX40+αPD1 regimen of *in situ* vaccination. The maximum suppression of distant graft development on day 14 was observed in the groups receiving injections of anti-OX40 antibodies: CpG+αOX40 and CpG+αOX40+αPD1 (***Fig. 5A***). Administration of two aptamers in combination with CpG ODNs has a noticeable antitumor effect for the A20 lymphoma model. It reduced the average tumor size (***Fig. 5A***) and the number of mice having a tumor at treatment site, compared with the control (*P*<0.05) (***Fig. 5B***).

A model for the assessment of tumor immunogenicity according to the ratio between the size of tumor grafts developed at the treatment site and the distant site was developed by Ruzanova *et al*^[58]^. We used this approach to evaluate the strength of immune response (an equivalent of immunogenicity induction) on day 14 upon *in situ* vaccination using all the experimental regimens. The results showed that a combination of CpG and two types of antibodies yielded the strongest immune response (***Fig. 5C***), meaning that tumor immunogenicity was a function of the regimens of *in situ* vaccination.

### Antitumor efficacy of *in situ* vaccination with CpG oligodeoxynucleotides, anti-OX40, and anti-PD1 antibodies and aptamers in the Ehrlich carcinoma model

Two series of experiments for the Ehrlich adenocarcinoma model were conducted. In the first instance, we evaluated the efficacy of the *in situ* vaccination for grafts transplanted in two hind paws of the same mouse, similar to the A20 lymphoma model. The efficacy of the *in situ* vaccination for two grafts simultaneously present in one mouse (an intramuscular graft and an ascites tumor graft) was determined at the second stage.

**Figure 6:**
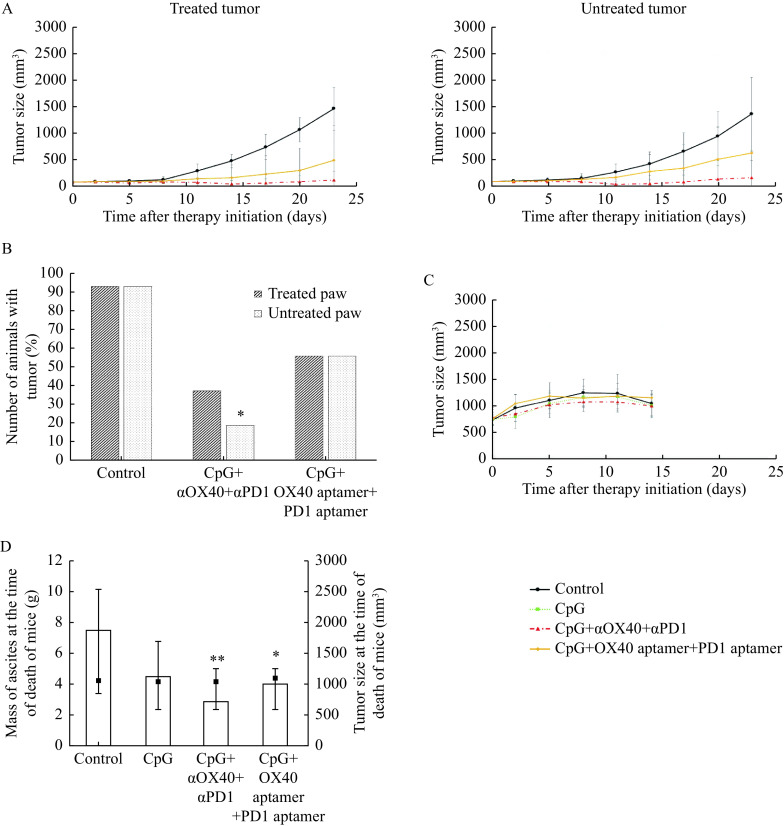
Efficacy of *in situ* vaccination for the Ehrlich carcinoma model.

Mice were intramuscularly engrafted with Ehrlich adenocarcinoma cells in both hind paws. CpG ODNs were injected in combination with anti-OX40 and anti-PD1 antibodies or aptamers. Among the studied groups, the maximum antitumor efficacy was observed for a vaccination with CpG+αOX40+αPD1 (*P*<0.05), compared with the control (***Fig. 6A***). *In situ* vaccination effectively slowed down tumor growth both at the treatment site and the distant site, unlike all the tumor models analyzed earlier. In this group, the tumor completely regressed at the treatment site and at the distant site in 60% and 80% of mice, respectively (***Fig. 6B***). Injection of CpG ODNs together with both OX40 and PD1 aptamers uniformly slowed down tumor growth, but nonsignificant, compared with the control (*P*˃0.05, ***Fig 6A*** and ***B***). The tumor completely regressed at both sites in 40% of animals (***Fig. 6B***). The efficacies of different antitumor therapy regimens for different tumor models are compared in ***Table 2***.

**Table 2:** Relative tumor size in different models that received antitumor therapeutic regimen treatment

Therapeutic regimens	Krebs-2 carcinoma (*n*=9)			Lewis carcinoma (*n*=7)			А20 B cell lymphoma (*n*=7)			Ehrlich carcinoma (*n*=5)	
	Treated (%)	Untreated (%)		Treated (%)	Untreated (%)		Treated (%)	Untreated (%)		Treated (%)	Untreated (%)
Oligo+αOX40	66.1	60.7		145.1	146.8		–	–		–	–
µCpG+αOX40	87.0	89.0		98.6	125.0		55.7	67.3		–	–
CpG+αOX40	30.1	35.9		24.4	51.5		2.4–3.2	32.0–36.5		–	–
CpG+αOX40+αPD1	34.6	37.8		24.6	53.7		0.0	38.0–48.4		7.1	10.7
CpG	–	–		–	–		9.0	80.9		–	–
CpG+OX40 aptamer	–	–		–	–		20.1	68.7		–	–
CpG+OX40 aptamer +PD1 aptamer	–	–		–	–		0.7	73.3		32.7	44.9
Mice were engrafted with two solid tumors. Oligodeoxynucleotides (ODNs) and antibodies or aptamers were injected intratumorally into the right paw three times every other day. Control animals were treated with saline according to the same regimen. Tumor in the right paw is referred to as "Treated" and the left paw as "Untreated".The values are the relations of the mean tumor size in the experimental group to that in the control group in percentages. They were calculated at different time points after receiving antitumor therapeutic regimen treatment (Krebs-2 carcinoma, day 16; Lewis carcinoma, day 12; А20 B cell lymphoma, day 11–14; Ehrlich carcinoma, day 23). For A20 B cell lymphoma the data are from two different experiments. Oligo: phosphorothioate non-CpG ODNs; µCpG: mesyl phosphoramidate CpG ODNs; CpG: phosphorothioate CpG ODNs.											

Mice were engrafted with Ehrlich adenocarcinoma cells intraperitoneally and intramuscularly in the right hind paw. Animal groups identical to those in the previous experiment were formed. Additionally, the group of mice that received CpG ODNs only was investigated. No significant reduction of solid tumor volume at the treatment site was revealed for all the study groups (*P*˃0.05, ***Fig. 6C***).

Injection of CpG+αOX40+αPD1 was found to be the most effective for ascites (***Fig. 6D***). In this group of mice, average ascites tumor weight at the time of animal's death was 2.6 times smaller, compared with the control group (*P*<0.01). Injections of CpG ODNs with OX40 and PD1 aptamers reduced average ascites tumor weight by 1.9 times, compared with the control (*P*<0.05). Injections of CpG ODNs alone reduced ascites tumor weight not significantly compared with the control (*P*˃0.05). Complete tumor resorption was attained in none of the study groups.

The results demonstrated that the injection of CpG and αOX40 potentiated by anti-PD1 antibodies had the strongest antitumor effect. Aptamers used instead of antibodies also exhibited antitumor activity, although it was less pronounced.

### Effects of *in situ* vaccination on heterologous tumors growing in the same organism in the models of A20 B cell lymphoma and Ehrlich carcinoma

**Figure 7:**
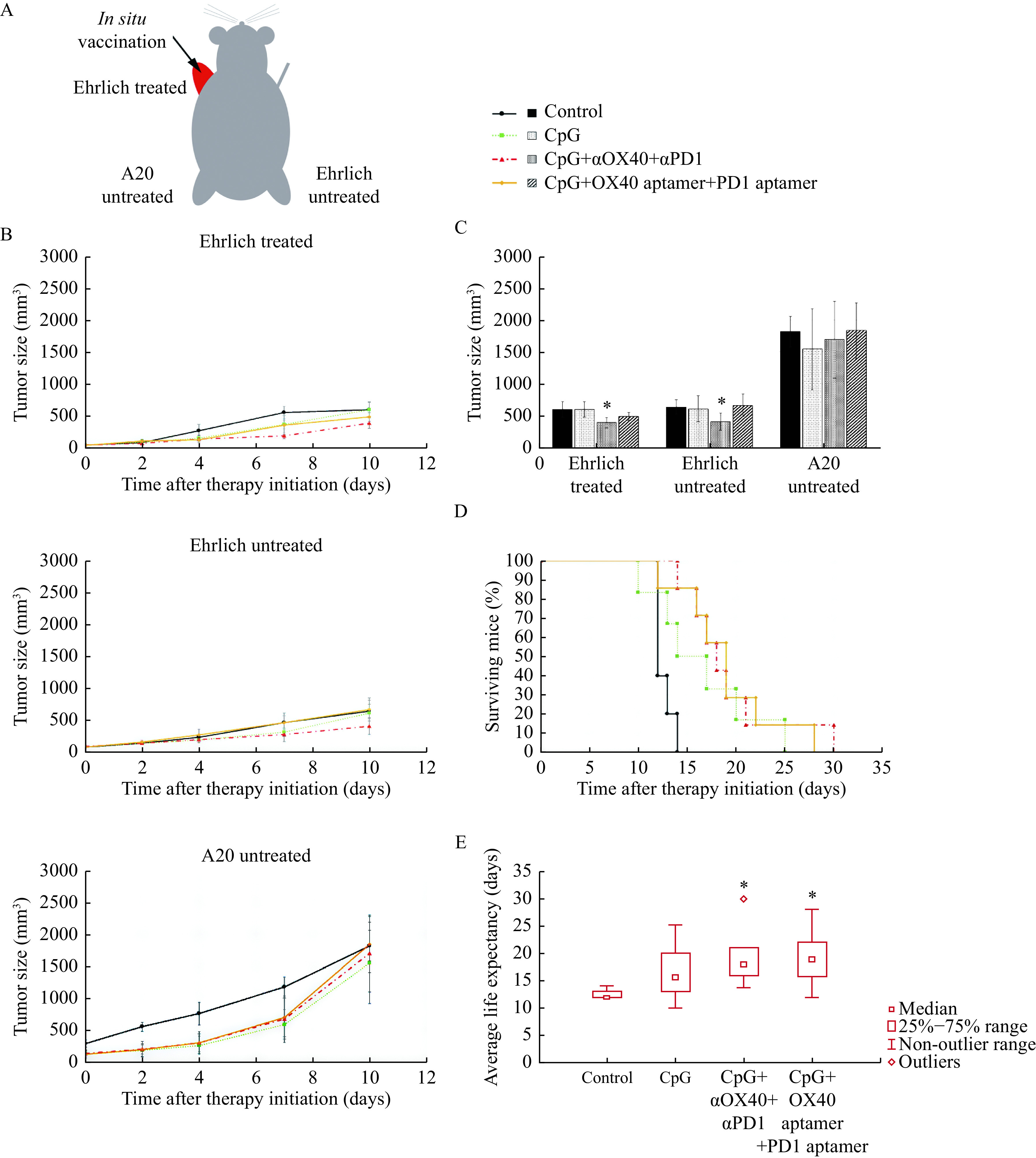
Efficacy of *in situ* vaccination for heterologous A20 B cell lymphoma and Ehrlich adenocarcinoma growing in the same organism.

To assess the antigenic specificity of the immune response elicited by *in situ* vaccination, two genetically distinct immunogenic tumors, A20 lymphoma and Ehrlich carcinoma, were grafted into the same mouse. Ehrlich carcinoma was grafted in the right hind and left front paws of mice. A20 lymphoma was simultaneously grafted in the left hind paw (***Fig. 7A***). The development of the immune response was evaluated by comparing the growth rates of the grafts in experimental mice to those growing at the same sites in control mice. CpG ODNs in combination with anti-OX40 and anti-PD1 antibodies or aptamers were injected. Mice receiving injections of saline or CpG ODNs only were used as controls. Injections were made intratumorally in the left front paw with Ehrlich adenocarcinoma graft.

The following evaluations were made: (a) whether the *in situ* impact on Ehrlich adenocarcinoma can elicit a tumor-specific immune response on the genetically distinct A20 B cell lymphoma; and (b) the efficacy of aptamers for the proposed regimen of *in situ* vaccination. Comparison of the development of homologous grafts at the treatment site and the distant site showed that *in situ* vaccination with CpG and αOX40 augmented by anti-PD1 antibodies activated the innate and adaptive immune responses. The results demonstrated that *in situ* vaccination with aptamers activated the system of innate immunity cells, while having no effect on eliciting the adaptive immune response. The activation of cross-protective adaptive immunity targeting A20 lymphoma was revealed for none of the vaccination regimens (***Fig. 7B*** and ***C***). *In situ* vaccination with antibodies and aptamers significantly increased (1.5-fold; *P*<0.05) the average survival time of the experimental mice (***Fig. 7D*** and ***E***).

## Discussion

In the current work, several modified variants of the *in situ* vaccination proposed by Sagiv-Barfi *et al*^[12]^ were analyzed. The first step of the current study was to investigate the ability of modified µCpG ODNs and OX40 aptamers to activate two types of immunocompetent cells involved in the induction and development of antitumor immunity, namely, DCs and cytotoxic T cells as the main therapeutic targets of technology. The modified µCpG ODNs showed high efficiency in *ex vivo* activation of DCs at a similar level as CpG or LPS. The effect of the aptamers was compared with conventional anti-OX40 antibodies. The possibility of using agonistic aptamers significantly simplifies the *in situ* vaccination procedure involving the use of monoclonal agonistic antibodies, which would also make it less expensive. Furthermore, the aptamer complex is non-toxic and does not elicit any checkpoint response. The ability to stimulate the maturation of naive T cells to effector T cells by OX40 aptamers at selected doses was also found to be comparable to conventional anti-OX40 antibodies.

During the second stage of the current study, an attempt was made to find specific conditions for factors chosen at the first stage, as well as some other known activators of specific immune responses that could potentiate the antitumor effect of the *in situ* vaccination approach described in Sagiv-Barfi *et al*^[12]^. Antibodies specific to OX40 co-stimulatory and PD1 checkpoint molecules were also added to the *in situ* vaccination procedure, supposing that the agonistic OX40 antibodies contribute to the enhancement of viability and potentiation of cytotoxic properties of effector T cells. Antagonistic PD1 antibodies inhibit the mechanism of T-cell anergy induction through the PD1 inhibitory molecule. Finally, we assessed the antitumor effect of OX40 and PD1 aptamers instead of specific anti-OX40 and anti-PD1 antibodies.

The OX40 receptor refers to T cell co-stimulatory molecules. The signaling pathway activated *via* the OX40/OX40L interaction is accompanied by T cell proliferation and cytokine secretion as well as an increase in T cell viability. In 2008, the multivalent OX40 RNA aptamers carrying two aptamer molecules hybridized to a single-stranded oligonucleotide template were synthesized, and their antitumor activity was characterized^[55]^. The two molecules of the aptamer dimerized head-to-tail in the complex with template were shown to simultaneously interact with two receptors, thereby resulting in receptor multimerization at the interaction site and T cell activation. In biological tests, the treating mice having experimentally aptamer-induced tumors led to tumor resorption.

When designing and synthesizing OX40 aptamers for the current study, we relied on Dollins *et al*^[55]^. To ensure the increased stability *in vivo*, the aptamers additionally contained two internucleotidic phosphorothioate groups at 3′- and 5′-ends, respectively, along with the 2′-deoxy-2′-fluorouridine and 2′-deoxy-2′-fluorocytidine residues. Similar to a foundational study by Dollins *et al*^[55]^, two molecules of the aptamer were dimerized on a flexible DNA template, which was also protected from enzymatic digestion by two phosphorothioate groups at the 3′- and 5′-ends and had a flexible *bis*-tetraethylene glycol phosphate spacer in the middle of the sequence. Therefore, two identical receptor binding sites were formed as a complementary duplex with a template in order to mimic the structure of the agonistic anti-OX40 antibody and attain the maximum efficiency of receptor binding.

Along with signaling through the OX40 co-stimulatory molecule, we intended to use an antagonistic molecule blocking T cell inhibition by PD1. Therefore, we synthesized a murine version of PD1 DNA aptamers, and the sequence of PD1 aptamer was taken from Prodeus *et al*^[56]^. To increase the bloodstream circulation time of PD1 aptamers, a palmitoyl group was attached to its 3′-end *via* the corresponding solid support, which is commercially available. Furthermore, to increase *in vivo* stability of the aptamer to nuclease digestion, the oligonucleotide was protected with three mesyl phosphoramidate (μ) groups at internucleotidic positions: two consecutive groups at the 3′-end including the palmitoylated abasic linker, and one at the 5′-end.

The main approach to assess the development of immune responses was to compare the development of tumor grafts at the treatment site and the distant site. The idea of this approach was first proposed in Sagiv-Barfi *et al*^[12]^ and was tested by us in Ruzanova* et al*^[58]^. It has been assumed that if tumor size at the treatment site decreases earlier than graft size at the distant site, therefore this is a response of the innate immune system. The subsequent reduction of the distant graft implies that the adaptive immune response is elicited. Here, we did not aim to trace all cellular and humoral components of immune reactions, since the approach proposed by Sagiv-Barfi *et al*^ [12]^ and further tested by us showed that this provides a suitable basis for assessment.

We showed for the tumors characterized by different immunogenicity that *in situ* vaccination strategy can be successfully used only for an immunogenic tumor, and in this experiment it was A20 lymphoma. We has additionally found that Ehrlich carcinoma is also a highly immunogenic tumor. Therefore, all the further experiments were conducted with two immunogenic tumors, specifically, A20 lymphoma and Ehrlich carcinoma.

Through experimentation, we found that μCpG exhibits no particular antitumor activity, when used for *in situ* vaccinations. This molecule was, therefore, excluded from further studies. These results are correlated with recent study in neonatal mice, when the splice-switching ability of mesyl phosphoramidate analog of nusinersen was shown to be inferior to the phosphorothioate counterpart^[60]^. The attachment of the 5′-palmitoyl group apparently had no beneficial effect on the *in vivo* activity of µCpG. The latter finding can be explained by numerous speculative considerations that remain outside the scope of this discussion. Nevertheless, it seems clear that experimental studies need to be continued to find a viable explanation for why there is no adequate response in *ex vivo* experiments.

The following regularities were revealed by comparing the efficacies of *in situ* vaccination with two types of antibodies and aptamers for the A20 lymphoma model. Administration of CpG+αOX40+αPD1 showed the highest antitumor efficacy among the studied groups. However, only this treatment reduced the size of the distant graft, thereby indicating that the adaptive immune response was elicited. All other treatments affect the target graft only, and the deceleration of its growth is related to the activation of the innate immune system. CpG ODNs were shown to have only insignificant antitumor activity, whereas the use of CpG ODNs in combination with two aptamers, OX40 and PD1, had a noticeably higher effect. All the studied vaccination regimens were compared according to immunogenicity induction for A20 lymphoma. Injection of CpG+αOX40+αPD1 had the strongest immunogenicity-inducing effect.

The *in situ* vaccination procedure was used for the experimentally induced Ehrlich carcinoma model. Two variants were studied: transplanting two grafts in two hind paws and transplanting a graft in the hind paw and a peritoneal ascites graft. In both cases, one hind paw was treated.

In the first variant, the maximum antitumor efficacy among all the studied groups was again observed for the combination of CpG+αOX40+αPD1. The inhibition of tumor growth at both sites observed after the injection of CpG+OX40 aptamer+PD1 aptamer was possibly related primarily to the adjuvant effect of CpG ODNs.

The second variant clearly demonstrated the antitumor effect of *in situ* vaccination with aptamers. Like the previous case, the impact on ascites was maximal for the injection of CpG+αOX40+αPD1. Injections of the CpG ODNs in combination with OX40 and PD1 aptamers statistically significantly reduced ascites tumor weight assessed at the time of animal's death. Complete ascite resorption was attained in none of the study groups. There was a statistically significant reduction in tumor size at the treatment site that was revealed in none of the study groups. It can be stated that for this tumor graft variant, the treatment activates adaptive immunity and is accompanied by a significant reduction of peritoneal ascites.

The following assessments were made in the final part of the study: (a) whether the development of tumor-specific immune response elicited by *in situ* vaccination targeting Ehrlich carcinoma can affect the genetically distinct A20 B cell lymphoma and (b) the efficacy of OX40 and PD1 aptamers for the proposed regimen of *in situ* vaccination. It turned out that only *in situ* vaccination with CpG and αOX40 augmented by addition of anti-PD1 antibodies elicits both innate and adaptive immune responses. The aptamers used for *in situ* vaccinations activated only innate immunity. The cross-protective adaptive immune response specific to A20 lymphoma was revealed for none of the regimens.

Our results demonstrate that *in situ* vaccination of a combination of phosphorothioate CpG ODNs and anti-OX40 antibodies potentiated by the addition of anti-PD1 antibodies exhibits the strongest antitumor effect both at the treatment site (the innate immunity) and at the distant site (the adaptive immunity). The respective OX40 and PD1 aptamers used instead of antibodies also showed an antitumor effect, although it was less pronounced. Additional biological studies are needed to optimize the doses of the aptamers required to ensure the maximum antitumor effect of these molecules.
